# Medical Opinion and Movement

**Published:** 1909-07-03

**Authors:** 


### Medical Opinion and Movement.

An interesting paper on the Functions of the Omentum was recently read before the Académie Royale de Médecine de Belgique by Heger and Heger-Gilbert. In a first series of experiments, a few c.c. of physiological salt solution, in which animal charcoal had been suspended, were injected into the peritoneal cavity of various animals. Care was taken to spread the solution over the whole of the peritoneum. The particles were subsequently found to have collected in the omentum, which had become thicker than normal. Microscopical examination showed the leucocytes, which were extremely abundant, to be engaged in an active phagocytosis of the charcoal particles. This action took place in both the lesser and greater omentum. In a second series of experiments metallic particles were used instead of charcoal, and a series of radiographic pictures taken. The photographs showed the gradual accumulation of the metal in the omentum, a fact which was subsequently verified post-mortem. On the other hand, in animals such as young rabbits, in which the omentum is ill-developed, the metallic particles remain scattered throughout the abdominal cavity. The same is true of animals—*e.g.* the frog and fish, in which the diaphragm is absent. In a third series of experiments larger foreign bodies, such as glass beads, particles of lead and cork were introduced. As a rule these were rapidly encysted by the omentum, which hypertrophied in their neighbourhood. The glass beads were in some cases seen to travel along the lymphatic channels and to accumulate near the great curvature of the stomach. Some even collected near the origin of the thoracic duct. Heavier foreign bodies, such as lead particles, do not migrate in this manner, but become encysted *in situ* by the omentum. These encysted bodies eventually leave the omentum, and may be found almost anywhere in the abdominal cavity, a fact which may explain the presence of "foreign bodies" sometimes found in the abdomen at autopsies. Occasionally the encysted bodies ulcerate through an adjoining coil of intestine and are passed per rectum, a general infection being prevented by omental adhesions. The authors were also able to show by experiments that a similar protective function to that of the omentum is possessed by the large ligaments of the female pelvis.

---

Autoserotherapy, a proceeding first advocated by Gilbert, consists in the subcutaneous injection into a patient of a few c.c. of his own pleural effusion, to stimulate the rapidity of resorption of the whole effusion. Schnutgen in the *Berliner Klinische Wochenschrifte*, describes the results he has obtained by this method of treatment, his conclusions being as follows. The method hastens the resorption of acute pleural effusions, which is completed in a couple of weeks in slight cases. It has a much more important, and, especially, a much more regular action in stimulating resorption than has ordinary exploratory puncture. It can be employed at the onset without any risk of the effusion increasing or relapsing, even when such inflammatory signs are present as ordinarily contra-indicate exploratory puncture. Immediately after its first application the quantity of urine passed increases, and may even become double. When tried in cases of sero-fibrinous pleurisy both of tuberculous and other origin, the results are excellent. Suppurative and hæmorrhagic pleurisies, as well as hydrothorax, with or without ascites, are in no way influenced by the treatment. The good effects obtained are due partly to the mechanical stimulus of the exploring needle, but in the main to the antitoxic and bactericidal bodies contained in the effusion, which are put into the circulation by this method. The author advises that 1 c.c. of the fluid should be withdrawn from the chest and injected subcutaneously. This is repeated every second day, and from three to six times in all, according to the severity of the case.

---

There appears in *L'Echo Médicale du Nord*, from the pen of Paquet, an article in which he discusses the Use of Blood-serum in the Treatment of the various types of Anæmia. Horse-serum was used in the cases upon which he bases his conclusions, this being administered either subcutaneously or by the mouth. Equally satisfactory results would seem to have ensued whichever method was adopted. The effect of the administration, as shown by blood examinations, was to increase rapidly the number of red corpuscles per cubic millimetre without, however, increasing the hæmoglobin index at a corresponding rate. An augmentation of red cells was also observed in healthy individuals submitted to a similar course of treatment; but this increase was quite transitory, which is in marked contrast with the results obtained in cases of chlorosis, where an increase of these cells above the normal was noted as late as two months after an injection. The forms of anæmia, in which the best results were obtained, are those of chlorosis, that following loss of blood from trauma, hæmatemesis, metrorrhagia, post-partum hæmorrhage, etc., and that following a severe illness or septic infection. The author has never found any dangers in the use of serum, such as fever, eruptions, and albuminuria, and believes that improvement follows more quickly by its use than by that of iron and arsenic. When administered subcutaneously two or three injections of 10 c.c. of serum, at intervals of two or three weeks, usually suffice to bring the blood-count up to the normal. If given by the mouth, 10 c.c. are administered daily for four consecutive days, the treatment being repeated at intervals of three weeks until a similar result is obtained.

---

At a recent meeting of the Medical Society of Vienna Dr. M. G. Schwartz reported on a means of preventing the harmful action of the X-rays on the tissues. He finds that the tissues are more sensitive to the *x*-rays when they are in a state of active metabolism. Seeds are not affected by the *x*-rays except when they are in a state of active vegetation. In the case of the skin, compression not only diminishes the circulation, but also inhibits metabolic changes. If an area of skin rendered ischæmic by compression is submitted to the action of the $x$-rays, there is produced only a transient hyperæmia, whereas in the adjacent area, not compressed, inflammation results. Further, the $x$-rays cause the hair to fall out, but if the skin is compressed the hair is unaffected. If, therefore, the skin is compressed much larger doses of the $x$-rays can be applied without fear of cutaneous complications. In support of these observations Dr. Nobl remarked that similarly the action of liquid carbolic acid is much more feeble on the skin, if it is compressed, than on the skin under ordinary conditions. The utility of these observations is quite obvious. By compressing the skin one should be able to apply the rays of greater strength, and, therefore, of more penetrating power in conditions in which it is desired to affect the deeper tissues, and also in cases of ringworm, for instance, one may be able to limit the field of action of the rays simply by compressing the surrounding skin.

---

MANY and various have been the treatments advocated for that troublesome condition of Furuncles, the most modern being the compression-cups of Bier. The following is the method advocated by Dr. G. Th. Jackson, Professor of Dermatology of the College of Physicians and Surgeons of New York. As soon as the furuncle begins to point, he pierces the summit with a pointed stick of wood dipped in 95 per cent. of carbolic acid. He does not use any pressure in the usual way to extrude the pus, but washes the surface around with hydrogen peroxide or with a 1-per-cent sublimate solution, and then applies gauze smeared with an ointment containing 5 to 10 per cent. salicylic acid. This is usually sufficient to effect a cure, but occasionally, if the furuncle is very large, the operation must be repeated the following day. The ointment must be kept applied for about a week. If fresh furuncles appear in the surrounding skin, due to infection before the application of the treatment, these must be treated in the same manner. If the furuncle fails to point, one or two drops of a 5 to 10 per cent. solution of carbolic acid should be injected and the ointment applied as already described. The author states that he has used this method for many years with complete success, and was taught this manner of treatment by Dr. G. H. Fox, previous Professor of Dermatology of the same college.

---

IN an interesting contribution to *La Semaine Médicale*, Dr. L. Cheinisse, of Paris, discusses the occurrence of Hæmorrhages in the Course of Influenza, and especially of Post-Influenzal Hæmorrhages. Although in recent years the occurrence of hæmorrhage in the course of the disease—epistaxis, hæmoptysis, and the like—has been recorded by various authors as pathological phenomena of an exceptional nature, the author shows that as far back as 1872 the description of the disease given in Jacoud's "Dictionnaire de Médecine et de Chirurgie Pratiques" includes these phenomena. But to go still further back, Etienne Pasquier in 1643 describes an epidemic disease analogous in all respects to influenza, in which he says there was a tendency to great effusions of blood by the mouth, the nose, and the rectum. Perhaps the most usual and best recognised form of hæmorrhage is the epistaxis, which often precedes or accompanies the onset of the disease. Morin distinguishes from this a form of hæmorrhagic influenza analogous to hæmorrhagic variola or hæmorrhagic measles, and again what he calls "critical hæmorrhage," when a pseudo-meningitic influenza suddenly clears up on the occurrence of a profuse epistaxis. Dr. Cheinisse's particular observations, however, refer to the ocurrence of hæmorrhages some time after an attack of the disease, and he records three cases in which more or less severe and obstinate epistaxis occurred some three or four weeks after the commencement of convalescence and without any apparent cause apart from the disease. He refers also to the report by Dr. Grainer of four cases of cerebral hæmorrhage following some time after attacks of influenza without any known constitutional predisposition. From these facts and observations the author seems justified in his conclusion that the disease carries with it a tendency to hæmorrhagic manifestations, and he suggests that they may be caused by a functional disturbance of the vaso-motor nerves leading to an excessive dilatation of the blood-vessels and consequent congestion.

---

BEURMANN, the discoverer of the Sporotrichium Beurmannii, contributes to the current number of the *Revue de Chirurgie* an interesting summary of the pathological lesions due to the fungus. Clinically the sporotrichium is important, because the lesions it causes are liable to be mistaken for tubercular deposits, gummata, or actinomycotic foci. Beurmann describes several cases in which bony and periosteal lesions were present, and in which a diagnosis of new growth or of an infective granuloma might reasonably have been made. In England the disease appears to be very rare, though cases of sporotrichosis affecting the skin have been recorded. In practice the differential diagnosis is very important, since lesions due to the fungus—which can always be fairly easily demonstrated microscopically—are singularly benign when properly treated. The prognosis in periosteal and old indurative cases is less good, but even here complete cures have been recorded in cases where the disease has existed for many months. Beurmann recommends fairly large doses of potassium iodide, giving as much as 90 grains of the salt per day. He also speaks highly of painting the surface of the lesion, especially where the skin is broken, with a lotion containing potassium iodide and tincture of iodine. This local treatment, combined with regular doses of the iodine internally, is usually effective in combating the disease.

---

THE marked infrequency of Tuberculous Peritonitis in association with Pregnancy, as compared with the quite common coincidence of other tuberculous lesions with the latter condition, is the subject of a paper by Dr. J. Pery, abstracted in the *Medical Review*. The explanation offered, probably with correctness, is that the adhesions and other changes produced cause sterility. It might also be suggested that the considerable number of cases in which the mischief starts as a tuberculous salpingitis may have something to do with the matter. In the case reported by this author, the diagnosis proved to be so extremely difficult that it was not until after tapping the abdomen that it was finally solved. The patient was in the fifth month of her fourth pregnancy when admitted to hospital suffering from abdominal pain of a fortnight's duration with vomiting, orthopnœa, and much distension of the belly. There was slight cough and profuse nocturnal sweats, but no hæmoptysis. Respirations were 50 per minute, and the pulse was small, irregular, and very rapid. In the epigastrium was a mass which gave the impression of an extremely distended uterine fundus. Percussion gave a line of dulness concave downwards, with a little resonance in the left flank undisturbed by change of posture. The diagnosis was considered doubtful between hydramnios and ascites, probably the former; but the result of tapping was to draw off more than five pints of blood-stained tuberculous fluid, after which the uterus could be made out distinct from other nodular masses. After a second tapping the patient aborted; she eventually left hospital with signs of double pleural effusion, as well as of tuberculous peritonitis, and has been lost sight of.

The prophylactic removal of the Appendix in every case of abdominal section in which there is no pressing necessity to conclude the operation at once finds an enthusiastic advocate in Dr. Lapthorn Smith, of Montreal. This surgeon has come round to the opinion that a laparotomy should be begun by inspection of the appendix, which should be removed if it cannot readily be brought up into the incision, or if, when brought up, it is found to be kinked by bands or adhesions. He would, in fact, like to lay down the removal of the viscus in all laparotomies, but is content for the present with the less sweeping procedure. He narrates cases in which intestinal obstruction and peritonitis, after various laparotomies, have been traced to appendicitis; and he also has a strong impression that many lesions diagnosed as puerperal fever really originate in a chronically inflamed appendix. In the discussion of this paper, which was read before the American Gynæcological Society, several operators expressed themselves as very averse to the removal of a healthy appendix when the abdomen is opened for some other operation. Dr. Peterson at one time removed two hundred consecutive appendices as a prophylactic measure, and found 50 per cent. of them to be microscopically diseased. Since then he has not removed the appendix unless it is macroscopically abnormal, and has had no reason to regret his withdrawal from the rather more advanced views he formerly held. The question is one which has more than one side to it; but probably few British surgeons practise what Dr. Lapthorn Smith preaches.

The Annual Report of Queen Charlotte's Lying-in Hospital contains, as usual, many interesting facts and figures from the year's record of cases. Antepartum Hæmorrhage was treated twenty-seven times out of one thousand eight hundred and sixty-five deliveries. Of thirteen cases of placenta prævia four were central, two marginal, and seven lateral: five of the patients went to term. Of the mothers two died, one of them from septicæmia; but of the children a much higher mortality was recorded, for but three survived. As for treatment, Champetier de Ribe's bag was used five times, version done thrice, forceps used four times, and plugging of cervix and vagina resorted to once. The other fourteen cases of hæmorrhage were all idiopathic: that is, no injury or fall had been sustained to account for the onset of the accidental hæmorrhage. Five cases were of severe type, but no maternal deaths were recorded: ten of the children died. From analysis of the urine of every patient admitted to the hospital it would appear that 17.5 per cent. of primipuræ show albuminuria at the time of delivery, and that 6.9 per cent. of multiparæ do so. An additional 6.9 per cent. of primiparæ and 5.7 per cent. of multiparæ present this symptom not only at delivery but until at least the fifth day of the puerperium also. Finally, percentages of 0.9 and 0.5 respectively are markedly albuminuric, with symptoms either of nephritis or eclampsia, or both. Another interesting table is that showing the details of all the cases of contracted pelvis treated in the hospital during the year. In view of the condemnation abroad by several leading obstetricians of the induction of premature labour as a means of treating these patients, it is satisfactory to see what excellent results the method yields in the hands of the Queen Charlotte's staff.

Mr. C. B. Lockwood contributes to the *Clinical Journal* some interesting remarks on Short Circuits in the Lymphatic Dissemination of Cancer in various situations. In dealing with carcinoma of the tongue, very early glandular deposit is especially to be looked for in a small lymph node which is embedded in the submaxillary salivary gland, especially when the disease is in the middle third of the tongue. He describes also another short circuit from this situation, along lymph vessels which cross the ala of the thyroid cartilage, loop upwards and outwards in the region of the cricoid to end in a gland just above the omo-hyoid muscle. In connection with the breast there is a short cut to the main deep lymph vessels by a trunk which does not traverse the lower axillary glands, but passes at first upwards and then through the pectoralis, or between that muscle and the deltoid, and empties into the lymphatics beneath the costo-coracoid membrane close to the subclavious muscle. When dealing with cancer of the external organs of generation, the crossed lymph channels must be borne in mind and the superficial and deep inguinal glands dealt with on both sides of the body, no matter how unilateral the lesion. But Mr. Lockwood believes that this is not enough, and that the internal and external retro-crural glands must also be extirpated, for deep inguinal lymphatics enter the femoral sheath and others pass up the spermatic cord or round ligament through the inguinal canal. These glands are dealt with through an incision similar to that for ligature of the external iliac artery.

